# Klotho Sensitivity of the Neuronal Excitatory Amino Acid Transporters EAAT3 and EAAT4

**DOI:** 10.1371/journal.pone.0070988

**Published:** 2013-07-29

**Authors:** Ahmad Almilaji, Carlos Munoz, Tatsiana Pakladok, Ioana Alesutan, Martina Feger, Michael Föller, Undine E. Lang, Ekaterina Shumilina, Florian Lang

**Affiliations:** 1 Department of Physiology, University of Tübingen, Tübingen, Germany; 2 Department of Psychiatry and Psychotherapy, University Psychiatric Clinics (UPK) Basel, Basel, Switzerland; Emory University, United States of America

## Abstract

Klotho, a transmembrane protein, which can be cleaved off as β-glucuronidase and hormone, is released in both, kidney and choroid plexus and encountered in blood and cerebrospinal fluid. Klotho deficiency leads to early appearance of age-related disorders and premature death. Klotho may modify transport by inhibiting 1,25(OH)_2_D_3_ formation or by directly affecting channel and carrier proteins. The present study explored whether Klotho influences the activity of the Na^+^-coupled excitatory amino acid transporters EAAT3 and EAAT4, which are expressed in kidney (EAAT3), intestine (EAAT3) and brain (EAAT3 and EAAT4). To this end, cRNA encoding EAAT3 or EAAT4 was injected into *Xenopus* oocytes with and without additional injection of cRNA encoding Klotho. EAAT expressing *Xenopus* oocytes were further treated with recombinant human β-Klotho protein with or without β-glucuronidase inhibitor D-saccharic acid 1,4-lactone monohydrate (DSAL). Electrogenic excitatory amino acid transport was determined as L-glutamate-induced current (I_glu_) in two electrode voltage clamp experiments. EAAT3 and EAAT4 protein abundance in the *Xenopus* oocyte cell membrane was visualized by confocal microscopy and quantified utilizing chemiluminescence. As a result, coexpression of Klotho cRNA significantly increased I_glu_ in both, EAAT3 or EAAT4-expressing *Xenopus* oocytes. Klotho cRNA coexpression significantly increased the maximal current and cell membrane protein abundance of both EAAT3 and EAAT4. The effect of Klotho coexpression on EAAT3 and EAAT4 activity was mimicked by treating EAAT3 or EAAT4-expressing *Xenopus* oocytes with recombinant human β-Klotho protein. The effects of Klotho coexpression and of treatment with recombinant human β-Klotho protein were both abrogated in the presence of DSAL (10 µM). In conclusion, Klotho is a novel, powerful regulator of the excitatory amino acid transporters EAAT3 and EAAT4.

## Introduction

Klotho is expressed in several tissues with particularly high expression in kidney and choroid plexus of the brain [1], [2]. The extracellular domain of the Klotho protein may be cleaved off and released into blood or cerebrospinal fluid and affect neighbouring cells as β-glucuronidase or hormone [3], [4]. Klotho-deficient mice suffer from severe growth retardation and premature appearance of a variety of age-related disorders resulting in death within less than 5 months [5], [6]. Conversely, the life span of mice is substantially extended by Klotho overexpression [5], [6].

Klotho is required for the inhibitory effect of FGF23 on 1α-hydroxylase and thus on 1,25(OH)_2_D_3_ formation [2], [6]–[8]. Functions of 1,25(OH)_2_D_3_ include up-regulation of renal Ca^2+^ and phosphate transport [9], [10]. Largely due to excessive 1,25(OH)_2_D_3_ formation, plasma Ca^2+^ [11] and phosphate [10] concentrations are increased in Klotho-deficient mice [2], [7], [8], leading to vascular calcification [12], [13] and growth deficit [2]. Beyond its impact on 1,25(OH)_2_D_3_ formation, Klotho may more directly influence transport processes, including Na^+^, phosphate cotransport [4], [14], Na^+^/K^+^ ATPase [15], Ca^2+^ channels [16] and renal outer medullary K^+^ channels [17].

Transport systems expressed in intestine, kidney and brain, include the excitatory amino acid transporter EAAT3, which is required for dicarboxylic amino acid absorption in intestine and reabsorption in renal proximal tubules [18], [19] as well as for cellular excitatory amino acid uptake at the blood-brain barrier [20], into neurons [21]–[28], into retinal ganglion cells [29] and into glial cells [30]–[33]. Excitatory amino acid uptake into cerebellar Purkinje cells is accomplished by the excitatory amino acid transporter EAAT4 [23], [25], [34].

Compromised excitatory amino acid uptake in the brain may result in excitotoxicity [35]. Deranged function of EAAT3 may further contribute to the pathophysiology of schizophrenia [28], [36]–[41], epilepsy [42]–[46] and hepatic encephalopathy [47]. Impaired function of EAAT4 has similarly been implicated in schizophrenia [36], [39].

The excitatory amino acid transporters EAAT3 and EAAT4 are regulated by phosphatidylinositide (PI)- 3-kinase signaling [29], [48]–[50], which is in turn sensitive to klotho [51].

To explore, whether Klotho participates in the regulation of the excitatory amino acid transporters EAAT3 and EAAT4, cRNA encoding EAAT3 or EAAT4 was injected into *Xenopus* oocytes either without or with additional injection of cRNA encoding Klotho. Moreover, EAAT3 or EAAT4-expressing oocytes were treated with recombinant human β**-**Klotho protein. To elucidate glutamate transport, glutamate-induced current was determined utilizing the two electrode voltage clamp and EAAT3 and EAAT4 protein abundance by confocal microscopy and chemiluminescence.

## Methods

### Animal Experiments

*Xenopus* Oocytes were explanted from adult *Xenopus Laevis* (NASCO, Fort Atkinson, USA). *Xenopus Laevis* frogs were anaesthesized by a 0.1% Tricain solution. After confirmation of anaesthesia and disinfection of the skin, a small abdominal incision was made and oocytes were removed, followed by closure of the skin with sutures. All animal experiments were conducted according to the German law for the welfare of animals and the surgical procedures on the adult Xenous laevis were reviewed and approved by the respective government authority of the state Baden-Württemberg (Regierungspräsidium) prior to the start of the study (Anzeige für Organentnahme nach §6).

### Constructs

For generation of cRNA constructs were used encoding Klotho [14], EAAT3 [52], [53] and EAAT4 [54]. The constructs were used for the generation of cRNA as described previously [55].

### Voltage Clamp in Xenopus Oocytes

*Xenopus* oocytes were prepared as previously described [56]. cRNA encoding EAAT3 or EAAT4 (10 ng) with or without additional 7 ng of cRNA encoding Klotho was injected on the first day after preparation of the *Xenopus* oocytes [57]. All experiments were performed at room temperature (about 22°C) 3 days after the injection. Two electrode voltage clamp recordings were performed at a holding potential of -60 mV [58]. Pipettes were filled with 3 M KCl and had resistances of 0.3–3.0 MΩ. The data were filtered at 10 Hz and recorded with a GeneClamp 500 amplifier, a DigiData 1300 A/D-D/A converter and the pClamp 9.2 software packages for data acquisition and analysis (Axon Instruments, Foster City, CA, USA) [55]. The oocytes were maintained at 17°C in ND96 solution containing 88.5 mM NaCl, 2 mM KCl, 1 mM MgC1_2_, 1.8 mM CaC1_2_, 5 mM HEPES, 0.11 mM tretracycline (Sigma, Schnelldorf, Germany), 4 µM ciprofloxacin (Sigma, Schnelldorf, Germany), 0.2 mM gentamycin (Refobacin©), 0.5 mM theophylline (Euphylong©) and 5 mM sodium pyruvate (Sigma, Schnelldorf, Germany), pH was adjusted to 7.5 by addition of NaOH [59]. The control superfusate ND96 contained 96 mM NaCl, 2 mM KCl, 1.8 mM CaCl_2_, 1 mM MgCl_2_ and 5 mM HEPES, pH 7.4. The flow rate of the superfusion was 20 ml/min, and a complete exchange of the bath solution was reached within about 10 s. L-glutamate was added to the solutions at a concentration of 2 mM unless otherwise stated. Where indicated, recombinant human β**-**Klotho protein (10, 30 or 50 ng/ ml, R&D Systems) and D-saccharic acid 1,4-lactone monohydrate (DSAL, 10µ M, Sigma, Schnelldorf, Germany) were added.

### Detection of EAAT Cell Surface Expression by Chemiluminescence

Oocytes were incubated with primary mouse monoclonal anti-EAAC1/EAAT3 antibody (diluted 1∶200, Invitrogen, USA) or with monoclonal anti-HA antibody conjugated to Horseradish Peroxidase (diluted 1∶500, Miltenyi Biotec, Germany) in order to determine HA-tagged EAAT4. Next, oocytes were incubated with secondary, HRP-conjugated sheep anti-mouse IgG antibody (for EAAT3; diluted 1∶1000, GE Healthcare, München, Germany). The individual oocytes were placed in 96 well plates with 20 µl of SuperSignal ELISA Femto Maximum Sensitivity Substrate (Pierce, Rockford, IL, USA) and chemiluminescence of single oocytes was quantified in a luminometer (Walter Wallac 2 plate reader, Perkin Elmer, Juegesheim, Germany) by integrating the signal over a period of 1 s. The results display normalized relative light units [60].

### Immunocytochemistry

The oocytes were fixed in 4% paraformaldehyde for at least 4 h at room temperature. After washing with PBS, the oocytes were cryoprotected in 30% sucrose, frozen in mounting medium and placed on a cryostat. Sections were collected at a thickness of 8 µm on coated slides and stored at −20°C. For immunostaining, the slides were dried at room temperature, fixed in aceton/methanol (1∶1), washed in PBS and blocked for 1h in 5% bovine serum albumin in PBS. The primary antibodies used were goat anti-EAAT3 antibody (for detection of EAAT3, diluted 1∶2500, Millipore Corporation, USA) or rat anti-HA antibody (for detection of EAAT4, diluted 1∶100, clone 3 F10, Roche, Switzerland). Incubation was performed in a moist chamber overnight at 4°C. In the case of EAAT3, binding of primary antibodies was visualised with a swine anti-goat conjugated Alexa488 antibody (diluted 1∶1000, Invitrogen, Molecular Probes, Eugene, OR, USA). For detection of EAAT4, a goat anti-rat conjugated Alexa488 antibody (diluted 1∶200, Invitrogen, Carlsbad, California, USA) was used. The oocytes were analyzed by a fluorescence laser scanning microscope (LSM 510, Carl Zeiss MicroImaging GmbH, Germany) with A-Plan 40×/0.25. Brightness and contrast settings were kept constant during imaging of all oocytes in each injection series [61].

### Statistical Analysis

Data are provided as means ± SEM, n represents the number of oocytes investigated. To avoid any bias from differences between oocyte batches, statistical comparisons were always made within batches of oocytes. Data were tested for significance using analysis of variance (ANOVA) or student’s unpaired t-test, as appropriate. Results with p<0.05 were considered statistically significant.

## Results

The present study explored whether Klotho influences the excitatory amino acid transporters EAAT3 and EAAT4. To this end, cRNA encoding EAAT3 or EAAT4 was injected into *Xenopus* oocytes with or without additional injection of cRNA encoding Klotho and the glutamate-induced current was taken as a measure of the electrogenic glutamate transport.

**Figure 1:**
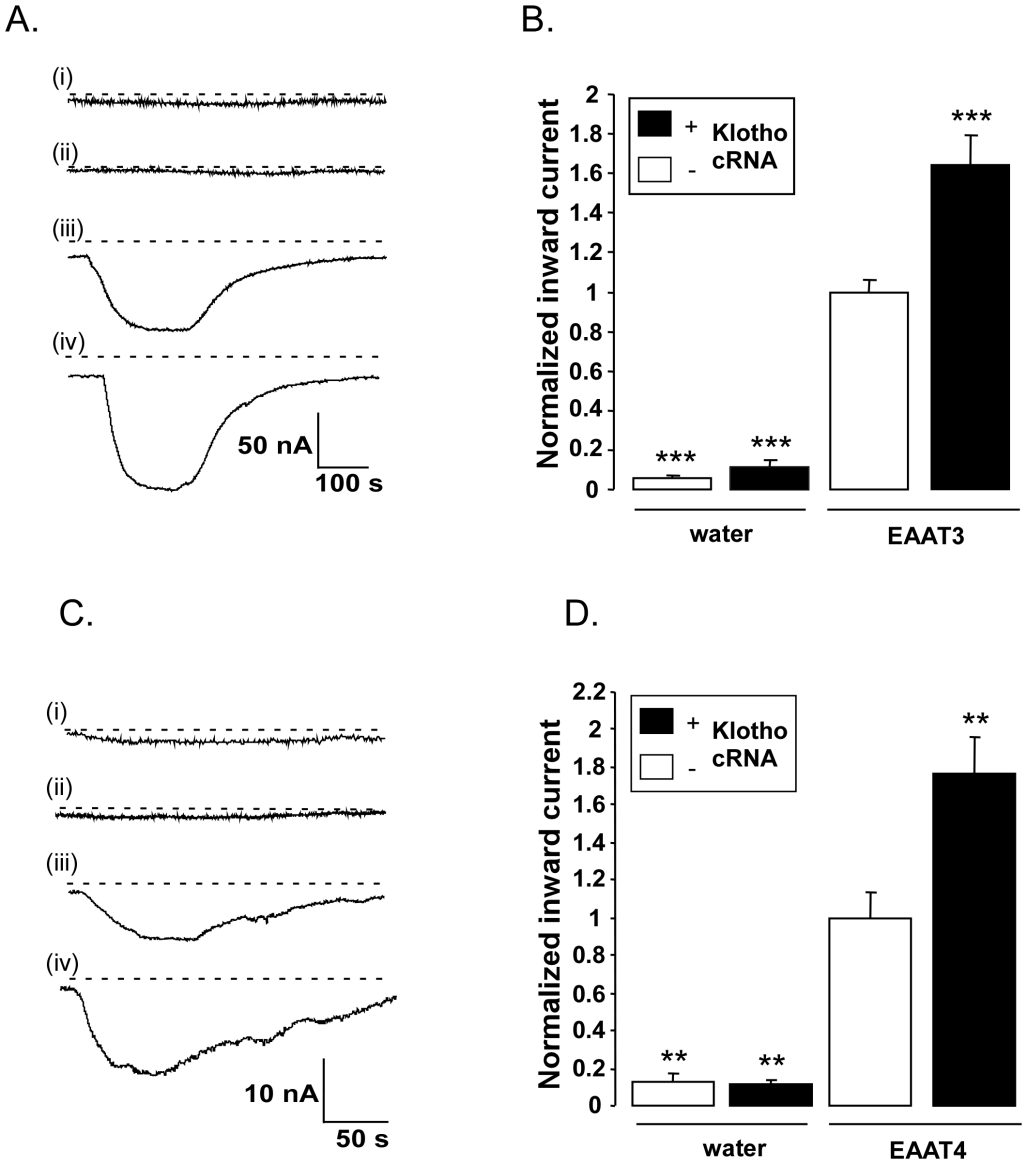
Effect of Klotho coexpression on electrogenic glutamate transport in EAAT3 or EAAT4 expressing *Xenopus* oocytes. **A:** Representative original tracings of glutamate (2 mM)-induced current (I_glu_) at −60 mV in *Xenopus* oocytes injected with water (i), or with cRNA encoding Klotho alone (ii), EAAT3 alone (iii) or both, EAAT3 and Klotho (iv). **B:** Means ± SEM (n = 7–36) of glutamate (2 mM)-induced current (I_glu_) in *Xenopus* oocytes injected without (left bars) or with (right bars) cRNA encoding EAAT3 and injected without (white bars) or with (black bars) cRNA encoding Klotho.***(p<0.001) indicates statistically significant difference from *Xenopus* oocytes injected with cRNA encoding EAAT3 alone (ANOVA). **C:** Representative original tracings of glutamate (2 mM)-induced current (I_glu_) measured at a holding potential of −60 mV in *Xenopus* oocytes injected with water (i), or with cRNA encoding Klotho alone (ii), EAAT4 alone (iii) or both EAAT4 and Klotho (iv). **D:** Means ± SEM (n = 5–8) of glutamate (2 mM)-induced current (I_glu_) in *Xenopus* oocytes injected without (left bars) or with (right bars) cRNA encoding EAAT4, and injected without (white bars) or with (black bars) cRNA encoding Klotho.**(p<0.01) indicate statistically significant difference from *Xenopus* oocytes injected with cRNA encoding EAAT3 or EAAT4 alone (ANOVA).

As illustrated in Fig. 1, negligible glutamate-induced current was observed in water-injected *Xenopus* oocytes or in oocytes injected with cRNA encoding Klotho alone. In contrast, the injection of cRNA encoding EAAT3 (Fig. 1 A,B) or EAAT4 (Fig. 1 C,D) was followed by the appearance of a marked inward current in the presence of glutamate. Additional injection of cRNA encoding Klotho led to a significant increase of the glutamate-induced current through EAAT3 (Fig. 1 A,B) and EAAT4 (Fig. 1 C,D).

**Figure 2:**
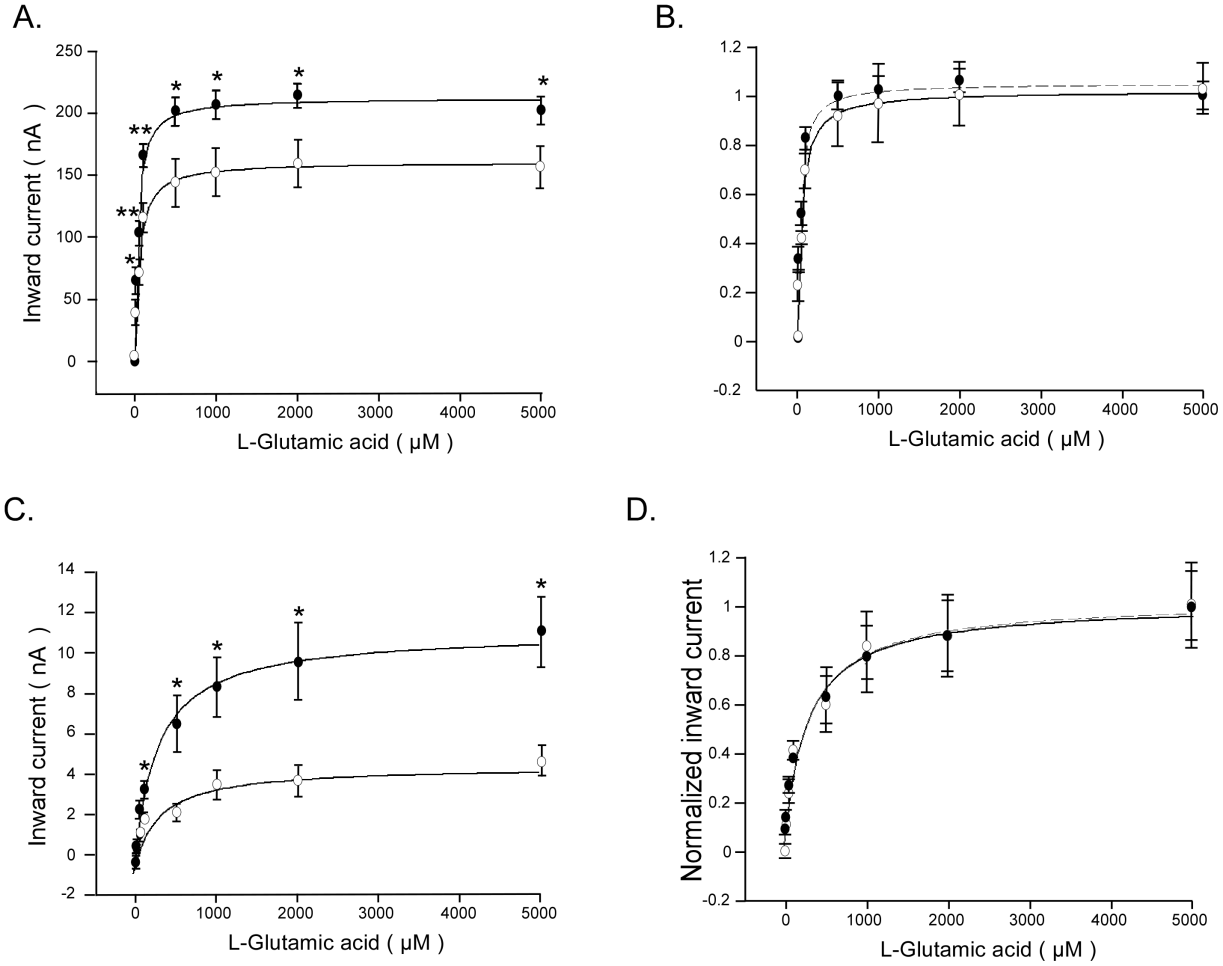
Glutamate-induced currents as a function of glutamate concentration in EAAT3/EAAT4-expressing *Xenopus* oocytes wihout or with Klotho coexpression. A, C: Means ± SEM of glutamate-induced current (I_glu_) as a function of glutamate concentration in *Xenopus* oocytes injected with cRNA encoding EAAT3 (A, n = 9) or EAAT4 (C, n = 6) without (open circles) or with (closed circles) additional coexpression of Klotho.*,**(p<0.05, p<0.01) indicate statistically significant difference from *Xenopus* oocytes injected with cRNA encoding EAAT3 (A) or EAAT4 (C) alone (two-tailed unpaired *t*-test). **B, D:** Means ± SEM of glutamate induced current (I_glu_) normalized to I_glu_ at 5 mM glutamate as a function of glutamate concentration in *Xenopus* oocytes injected with cRNA encoding EAAT3 (**B**, n = 9) or EAAT4 (**D**, n = 6) without (open circles) and with (closed circles) additional coexpression of Klotho. The values were fitted to a hyperbola function.

Kinetic analysis of glutamate induced currents was performed to elucidate whether Klotho coexpression modifies the affinity of the carriers. As illustrated in Fig. 2, the glutamate-induced current increased as a function of the substrate concentration. The maximal glutamate-induced current was significantly (p<0.05) higher in *Xenopus* oocytes injected with cRNA encoding both EAAT3 and Klotho (212.8±7.1 nA, n = 9) than in *Xenopus* oocytes injected with cRNA encoding EAAT3 alone (161.3±4.3 nA, n = 9) (Fig. 2 A). Similarly, the maximal glutamate induced current was significantly (p<0.05) higher in *Xenopus* oocytes injected with cRNA encoding both EAAT4 and Klotho (11.2±0.4 nA, n = 6) than in *Xenopus* oocytes injected with cRNA encoding EAAT4 alone (5.0±0.3 nA, n = 6) (Fig. 2C). The glutamate concentration required for half maximal glutamate-induced current was not significantly different (p = 0.1815) between *Xenopus* oocytes injected with cRNA encoding both EAAT3 and Klotho (34.5±6.9 µM, n = 9) and in *Xenopus* oocytes injected with cRNA encoding EAAT3 alone (48.7±7.4 µM, n = 9). Similarly, the glutamate concentration required for halfmaximal glutamate induced current was not significantly (p = 0.9236) different between *Xenopus* oocytes injected with cRNA encoding both EAAT4 and Klotho cRNA (274.3±48 µM, n = 6) and in *Xenopus* oocytes injected with cRNA encoding EAAT4 alone (283.3±78.2 µM, n = 6). It should be pointed out, however, that the scatter of the data precludes safe conclusions regarding effects of klotho on affinity of the glutamate carriers.

**Figure 3:**
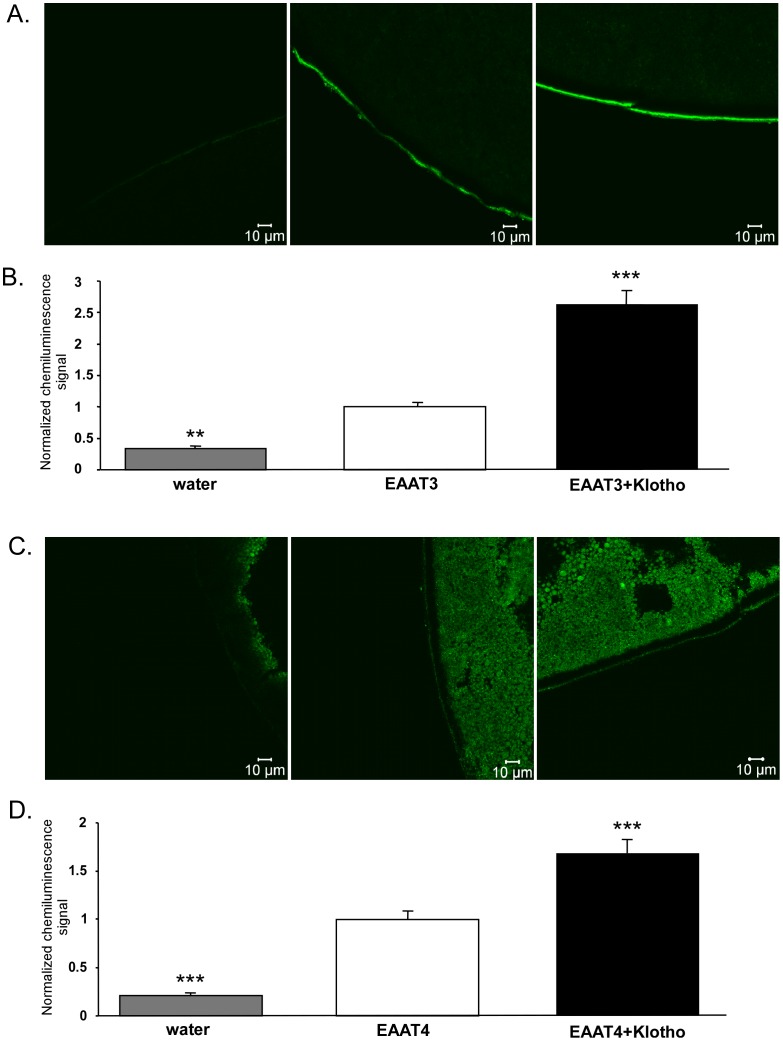
Effect of Klotho coexpression on protein abundance of both EAAT3 and EAAT4 in the *Xenopus* oocyte cell membrane. **A, C:** Confocal images of EAAT3 (A) and EAAT4 (C) protein abundance in the plasma membrane of *Xenopus* oocytes injected with water (1^st^ panel), injected with cRNA encoding EAAT3 (A) or EAAT4 (C) without (2^nd^ panel) or with additional coexpression of Klotho (3^rd^ panel**)**. **B, D:** Means ± SEM of EAAT3 (**B**, n = 75–80) and EAAT4 (**D**, n = 82–87) protein abundance as determined by chemiluminescence in the plasma membrane of *Xenopus* oocytes injected with cRNA encoding EAAT3 (**B**) or EAAT4 (**D**) without (white bars) or with (black bars) coexpression of Klotho. For comparison, water injected oocytes (grey bars).**,***(p<0.01, p<0.001) indicate statistically significant difference from *Xenopus* oocytes injected with cRNA encoding EAAT3/EAAT4 alone (ANOVA).

The increased maximal transport rate upon Klotho coexpression could have been due to an increase of EAAT3/EAAT4 protein abundance in the cell membrane. Confocal microscopy and chemiluminescence were thus employed in order to determine the EAAT3/EAAT4 protein abundance in the cell membrane of *Xenopus* oocytes. As illustrated in Fig. 3, injection of cRNA encoding Klotho significantly enhanced the EAAT3 (Fig. 3 A,B) and EAAT4 (Fig. 3 C,D) protein abundance in the cell membrane of oocytes injected with cRNA encoding EAAT3 or EAAT4.

**Figure 4:**
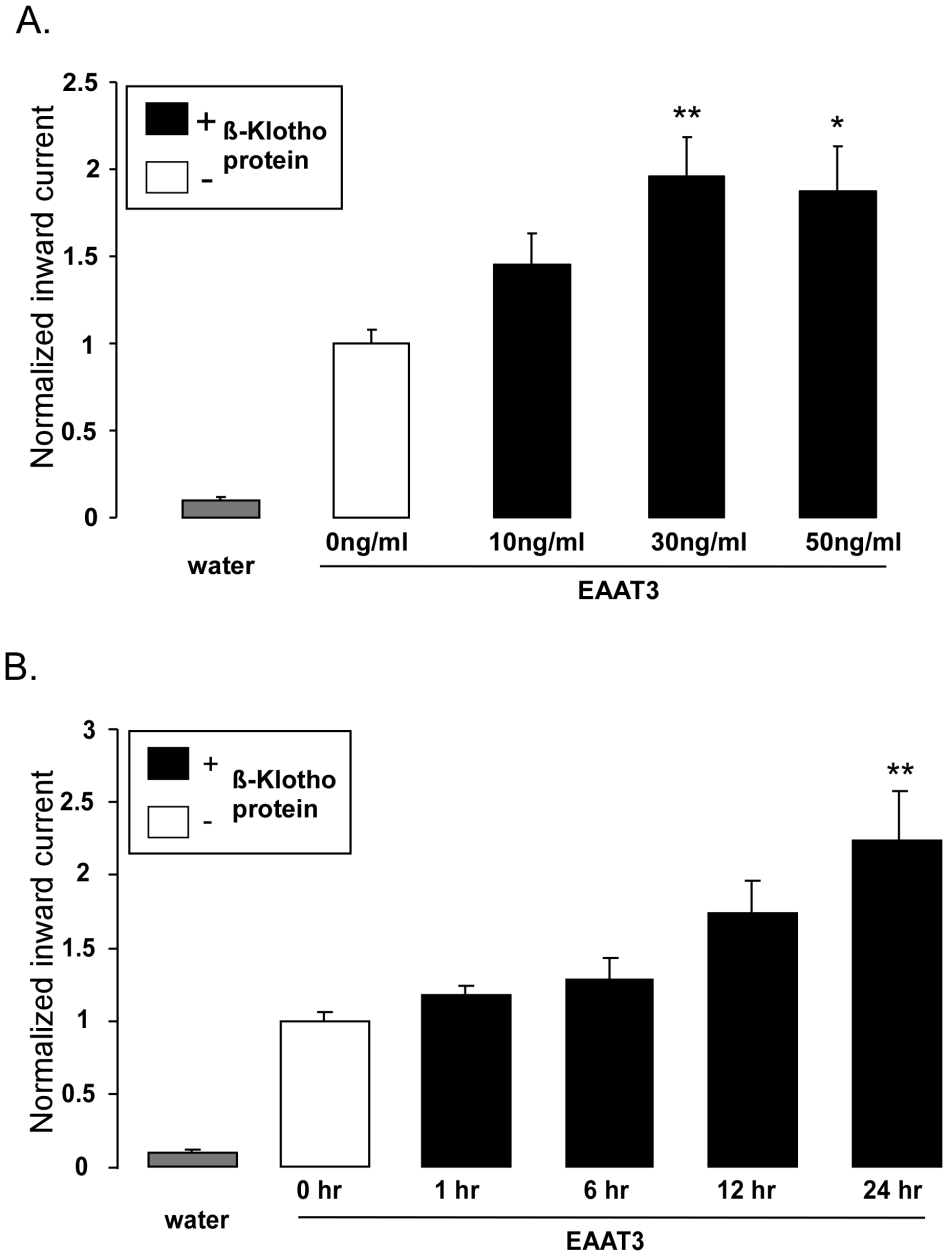
Effect of recombinant human β-Klotho protein on electrogenic glutamate transport in EAAT3 -expressing *Xenopus* oocytes. **A:** Means ± SEM (n = 7–16) of glutamate (2 mM)-induced current (I_glu_) in *Xenopus* oocytes injected with water (grey bar) or injected with cRNA encoding EAAT3 and pretreated prior to measurements for 24 hours without (white bar) or with 10, 30 and 50 ng/ml recombinant human β-Klotho protein (1^st,^ 2^nd^ and 3^rd^ black bar respectively).*,**(p<0.05, p<0.01) indicate statistically significant difference from untreated *Xenopus* oocytes (ANOVA). **B:** Means ± SEM (n = 12–17) of glutamate (2 mM)-induced current (I_glu_) in *Xenopus* oocytes injected with water (grey bar) or injected with cRNA encoding EAAT3 pretreated prior to measurements with 30 ng/ml recombinant human β-Klotho protein for 0 hr (white bar) or 1, 6, 12 or 24hr (black bars respectively).**(p<0.01) indicates statistically significant difference from untreated *Xenopus* oocytes (ANOVA).

Further experiments explored whether the effect of Klotho coexpression was mimicked by the pretreatment of EAAT3-expressing *Xenopus* oocytes with recombinant human β-Klotho protein. As shown in Fig. 4A, pretreatment of *Xenopus* oocytes injected with cRNA encoding EAAT3 with recombinant human β-Klotho protein (10, 30 and 50 ng/ ml) for 24 hours was followed by a gradual increase in the glutamate-induced inward current, an effect reaching statistical significance at the concentration of 30 ng/ ml. The effect of recombinant human β-Klotho protein (30 ng/ml) on the glutamate induced current of oocytes injected with cRNA encoding EAAT3 was time-dependent and reached statistical significance after 24 hours of treatment (Fig. 4B). Accordingly, in the next series of experiments β-Klotho protein was used at a concentration of 30 ng/ ml and an incubation time of 24 hours.

**Figure 5:**
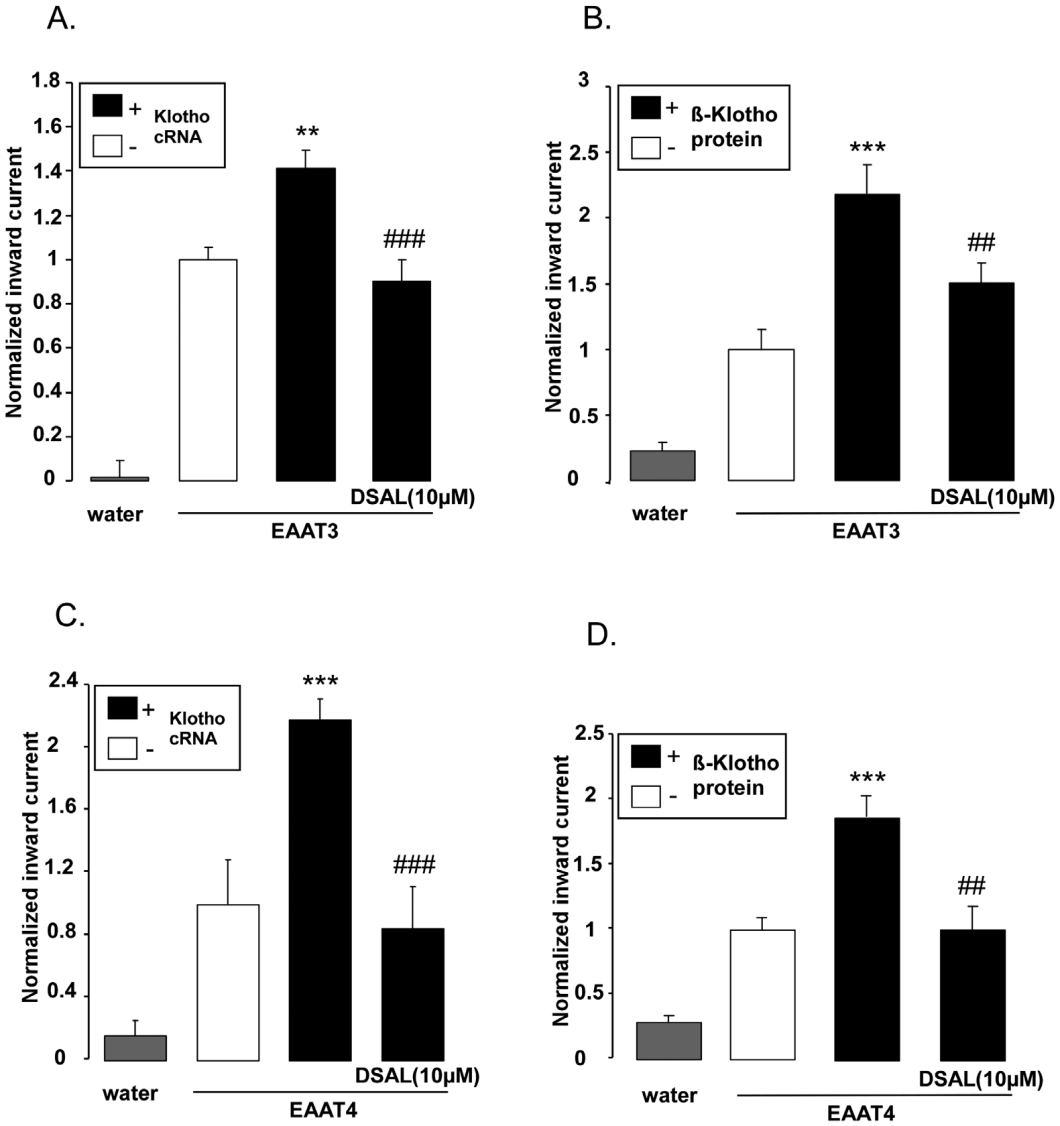
Reversal of the effect of Klotho on electrogenic glutamate transport in EAAT3 or EAAT4 expressing *Xenopus* oocytes by β-glucuronidase inhibitor DSAL. **A:** Means ± SEM (n = 9–21) of glutamate (2 mM)-induced current (I_glu_) in *Xenopus* oocytes injected with water (grey bar) or injected with cRNA encoding EAAT3 alone (white bar) or both EAAT3 and Klotho (black bars). Where indicated, the oocytes were treated with β-glucuronidase inhibitor DSAL (10 µM).**(p<0.01) indicates statistically significant difference from oocytes injected with cRNA encoding EAAT3 alone (ANOVA). ^###^ (p<0.001) indicates statistically significant difference from oocytes injected with cRNA encoding both EAAT3 and Klotho (ANOVA).**B:** Means ± SEM (n = 11–23) of glutamate (2 mM)-induced current (I_glu_) in *Xenopus* oocytes injected with water (grey bar) or injected with cRNA encoding EAAT3 alone (white bar) or pretreated for 24 hours with 30ng/ ml recombinant human β-Klotho protein without (first black bar) or with the presence of β-glucuronidase inhibitor DSAL (10µM ) (second black bar).***(p<0.001) indicates statistically significant difference from non-treated oocytes injected with cRNA encoding EAAT3 alone (ANOVA). ^##^(p<0.01). indicates statistically significant difference from oocytes injected with cRNA encoding EAAT3 and treated for 24 hours with 30ng/ ml recombinant human β-Klotho protein (ANOVA).**C:** Means ± SEM (n = 5–23) of glutamate (2 mM)-induced current (I_glu_) in *Xenopus* oocytes injected with water (grey bar) or injected with cRNA encoding EAAT4 alone (white bar) or both EAAT4 and Klotho (black bars). Where indicated, the oocytes were treated with β-glucuronidase inhibitor DSAL (10 µM).***(p<0.001) indicates statistically significant difference from oocytes injected with cRNA encoding EAAT4 alone (ANOVA). ^###^(p<0.001) indicates statistically significant difference from oocytes injected with cRNA encoding both EAAT4 and Klotho (ANOVA).**D:** Means ± SEM (n = 9–21) of glutamate (2 mM)-induced current (I_glu_) in *Xenopus* oocytes injected with water (grey bar) or injected with cRNA encoding EAAT4 alone (white bar) and pretreated for 24 hours with 30 ng/ ml recombinant human β-Klotho protein without (first black bar) or with the presence of β-glucuronidase inhibitor DSAL (10µM, second black bar).***(p<0.001) indicates statistically significant difference from non-treated oocytes injected with cRNA encoding EAAT4 alone (ANOVA). ^##^(p<0.01). indicates statistically significant difference from oocytes injected with cRNA encoding EAAT4 and treated for 24 hours with 30 ng/ml recombinant human Klotho protein (ANOVA).

An additional series of experiments explored whether the effect of Klotho was related to its β-glucuronidase activity. To this end, *Xenopus* oocytes, which were injected with cRNA encoding both, EAAT3 and Klotho (Fig. 5A) or both, EAAT4 and Klotho (Fig. 5C), were treated with the β-glucuronidase inhibitor DSAL (10 µM) for 24 hours prior the measurement. As illustrated in Fig. 5A, C, pretreatment of *Xenopus* oocytes with DSAL (10µM) abrogated the effect of Klotho encoding cRNA injection on glutamate-induced inward current of oocytes injected with cRNA encoding EAAT3 (Fig. 5A) and EAAT4 (Fig. 5C). Similarly, parallel pretreatment with β-glucuronidase inhibitor DSAL (10 µM) for 24 hours abrogated the effect of recombinant human β-Klotho protein (30 ng/ ml) on glutamate induced inward current of oocytes injected with cRNA encoding EAAT3 (Fig. 5B) and EAAT4 (Fig. 5D).

## Discussion

The present observations uncover a completely novel function of Klotho, i.e. the up-regulation of the excitatory amino acid transporters EAAT3 and EAAT4. Klotho increased the carrier protein abundance in the cell membrane and thus enhanced the maximal transport rate of the carriers. The effect apparently required the hydrolysis of β-D-glucuronic acid by Klotho, as it was reversed by the β-glucuronidase inhibitor. The effect of klotho on EAAT3 and EAAT4 contrasts the effect of klotho on Na^+^ coupled phosphate transporter NaPiIIa and NaPiIIb, which are both donwregulated by klotho [14].

Klotho further up-regulates the Na^+^/K^+^ATPase [15], [62], which is required to maintain the chemical gradient for Na^+^ coupled transport [63]. Thus, Klotho modifies excitatory anino acid transport not only by up-regulating the carrier protein, but at least in theory by additional maintaining the electrochemical gradient for Na^+^.

The effect of Klotho on EAAT3 may contribute to the regulation of renal tubular amino acid transport. In the kidneys, the excitatory amino acid transporter EAAT3 accomplishes dicarboxylic amino acid reabsorption in renal proximal tubules [19] and defective EAAT3 leads to dicarboxylic aminoaciduria [18]. Whether Klotho deficient mice suffer from amino aciduria is – to the best of our knowledge – not known. Dicarboxylic amino aciduria is expected only, if the lack of Klotho decreases the maximal transport rate of EAAT3 below the filtered load.

In the brain, EAAT3 contributes to excitatory amino acid transport at the blood-brain barrier [20], and to the clearance of excitatory amino acids from synaptic clefts by cellular uptake into neurons [21]–[28], retinal ganglion cells [29] and glial cells [30]–[33]. EAAT4 accomplishes excitatory amino acid transport into cerebellar Purkinje cells [23], [25], [34]. Decreased cerebral or cerebellar excitatory amino acid uptake in the brain is expected to cause excitotoxicity [35], [64]. Acccordingly, impaired function of EAAT3 may lead to epilepsy [42]–[46] and hepatic encephalopathy [65]. Moreover, deranged cellular excitatory amino acid uptake by EAAT3 [28], [36]–[41] or EAAT4 [36], [39] may foster the development of schizophrenia. Evidence for a role of glutamatergic neurotransmission in the pathophysiology of psychiatric disorders comes from studies using magnetic resonance spectroscopy, a technique that non-invasively measures in vivo concentrations of glutamate and other amino acids under different experimental conditions [66]. Morover, recent clinical studies have demonstrated that a single subpsychotomimetic dose of ketamine, an ionotropic glutamatergic N-methyl-D-aspartate (NMDA) receptor antagonist, produces a rapid antidepressant response in patients with major depressive disorder, with effects lasting up to 2 weeks [67]. Along those lines, altered EAAT expression has been found in schizophrenic and bipolar patients in frontal and temporal brain regions [40], [68]–[70]. Furthermore, administration of riluzole, a drug that enhances glutamate uptake through EAATs, reverses stress-induced motivational deficits and restores prefrontal BDNF expression after corticosterone [71]. Because riluzole has antidepressant effects in both, animal models and human subjects, it may represent the prototype for a novel class of antidepressants with the modulation of glial physiology as a primary mechanism of action [72].

Klotho deficiency has been shown to foster the degeneration of mesencephalic dopaminergic neurons leading to decreased levels of striatal dopamine [73]. The effect was, however, reversed by vitamin D restriction [73] and is thus presumably not the result from direct regulation of excitatory amino acid transport. Lack of Klotho expression further leads to cognitive deficits [65]. Klotho induces maturation of rat primary oligodendrocytic progenitor cells, an effect attributed in part to stimulation of Akt and ERK [47]. Klotho deficiency leads to a decrease of major myelin protein expression due to a decreased number of total and mature oligodendrocytes [65]. Klotho is downregulated in the aged brain, which is paralleled by decrease of white matter and myelin abnormalities [38], [74]. Whether or not oligodendrocyte maturation and survival is modified by the abundance of extracellular excitatory amino acids and thus by EAAT3 and EAAT4 activities, remains to be shown. Klotho abundance is downregulated by TNFα and thus, deranged expression of Klotho may participate in the pathophysiology of neuroinflammation [75]. It is tempting to speculate that Klotho sensitivity of EAAT3 and EAAT4 contributes to neurodegeneration during neuroinflammation. Clearly, additional studies will be required, however, to define the *in vivo* relevance of Klotho-sensitive excitatory amino acid transport.

In conclusion, the anti-aging protein Klotho up-regulates the excitatory amino acid transporters EAAT3 and EAAT4, an effect which may participate in the regulation of renal tubular transport of dicarboxylic amino acids and the clearance of excitatory amino acids from synaptic clefts in the brain. Mechanisms regulating glutamate cycling and metabolism including Klotho may be viable drug targets for depression and schizophrenia.
